# Deficiency of AXL in Bone Marrow-Derived Cells Does Not Affect Advanced Atherosclerotic Lesion Progression

**DOI:** 10.1038/srep39111

**Published:** 2016-12-13

**Authors:** Manikandan Subramanian, Jonathan D. Proto, Glenn K. Matsushima, Ira Tabas

**Affiliations:** 1 Department of Medicine, Columbia University Medical Center, New York, USA; 2 CSIR - Institute of Genomics and Integrative Biology, New Delhi, India; 3 Department of Microbiology & Immunology, UNC Neuroscience Center, Integrative Program in Biological Genome Sciences, University of North Carolina, Chapel Hill, NC, USA; 4 Department of Pathology and Cell Biology, Columbia University Medical Center, New York, USA; 5 Department of Physiology and Cellular Biophysics, Columbia University Medical Center, New York, USA

## Abstract

AXL, a member of the TAM (Tyro3, Axl, MerTK) family of receptors, plays important roles in cell survival, clearance of dead cells (efferocytosis), and suppression of inflammation, which are processes that critically influence atherosclerosis progression. Whereas MerTK deficiency promotes defective efferocytosis, inflammation, and plaque necrosis in advanced murine atherosclerosis, the role of Axl in advanced atherosclerosis progression is not known. Towards this end, bone marrow cells from *Axl*^*−/−*^ or wild-type mice were transplanted into lethally irradiated *Ldlr*^*−/−*^ mice. These chimeric mice were then fed the Western-type diet (WD) for 17 weeks. We demonstrate that lesional macrophages in WT mice express Axl but that Axl deficiency in bone marrow-derived cells does not affect lesion size, cellularity, necrosis, or inflammatory parameters in advanced atherosclerotic plaques. Moreover, apoptosis of lesional cells was unaffected, and we found no evidence of defective lesional efferocytosis. In contrast to previously reported findings with MerTK deficiency, hematopoietic cell-Axl deficiency in WD-fed *Ldlr*^*−/−*^ mice does not affect the progression of advanced atherosclerosis or lesional processes associated with TAM receptor signaling. These findings suggest a heretofore unappreciated TAM receptor hierarchy in advanced atherosclerosis.

Atherosclerosis is a chronic inflammatory disease of the vascular wall induced by apoB-containing lipoproteins deposited beneath the endothelium of large and medium-sized arteries^1^. While the vast majority of atherosclerotic lesions remain clinically silent, a small proportion undergo plaque rupture or erosion, which can precipitate acute thrombotic vascular occlusion and its consequences, *e.g*., myocardial infarction, unstable angina, sudden cardiac death, and stroke^2^,^3^. Thus, the molecular mechanisms involved in the conversion of benign to vulnerable plaques is an area of immense importance. Previous studies have implicated chronic non-resolving inflammation, lesional cell apoptosis, defective efferocytosis, plaque necrosis, and thinning of the fibrous cap as critical factors influencing this transition^2^,^3^.

The TAM family of receptors are increasingly being recognized to play a critical role in tempering inflammation in several chronic inflammatory as well as autoimmune disorders^4^. Signaling through these receptors has been shown to increase cell survival and promote efferocytosis with consequent generation of anti-inflammatory cytokine mediators^4^. Moreover, MerTK triggers an inflammation resolution signaling pathway in macrophages^5^. We and others have previously shown a critical role for MerTK, which is expressed primarily on macrophages, in promoting efferocytosis and suppressing inflammation in advanced atherosclerosis, with a net effect of preventing the expansion of necrotic areas in lesions^6^,^7^. However, the role of Axl, the other major TAM family member, in atherosclerosis progression is not known. In this context, a recent study demonstrated that treatment of cultured macrophages with inducers of inflammation is associated with an increase in Axl expression concomitant with a decrease in MerTK expression^8^. These data raise the possibility that Axl signaling may play a dominant role in efferocytosis and suppression of inflammation in pro-inflammatory environments such as that seen in advanced atherosclerotic plaques.

To examine this issue, we transplanted bone marrow cells from *Axl*^*−/−*^ mice into lethally irradiated *Ldlr*^*−/−*^ mice to generate chimeric mice that are deficient in Axl in all hematopoietic-derived cells including atherosclerotic lesional macrophages and dendritic cells. In this model, we demonstrate that lesional cells in wild-type mice express Axl but that deficiency of Axl does not affect atherosclerotic lesion size, lesional cell apoptosis, efferocytosis, plaque necrosis, inflammation, or fibrosis. These data suggest that Axl in bone marrow-derived cells does not play a significant role in advanced atherosclerotic plaque progression, which, in view of the important role of MerTK in plaque progression, indicates a fascinating TAM receptor hierarchy in advanced atherosclerosis.

## Results

**Figure 1:**
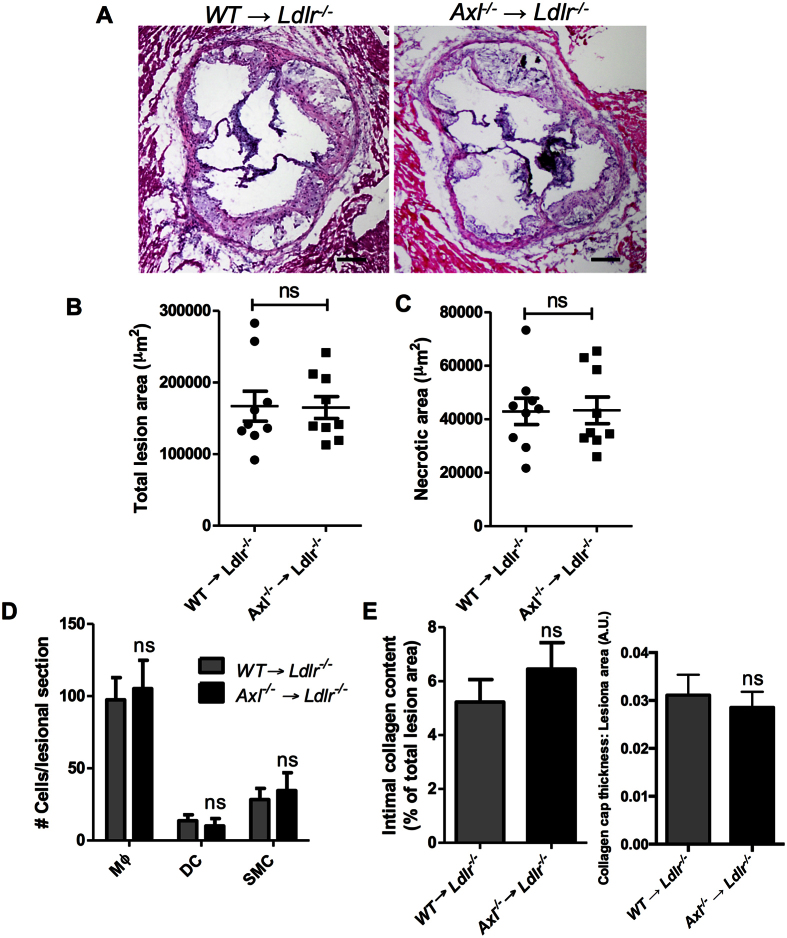
Hematopoietic cell-Axl deficiency does not affect advanced atherosclerosis progression. (**A**) Representative images of H&E-stained aortic root sections from WT → *Ldlr*^*−/−*^ or *Axl*^*−/−*^ → *Ldlr*^*−/−*^ mice fed the WD for 17 weeks. Bar, 200 μm. (**B**,**C**) Quantification of total atherosclerotic lesion area and necrotic area in the two groups of mice. (**D**) Quantification of cellular distribution in atherosclerotic lesions as characterized by immunostaining for F4/80 (macrophages, Mϕ), CD11c (dendritic cells, DC), and a-smooth muscle actin (smooth muscle cells, SMC). (**E**) Quantification of collagen content and cap thickness in the intima of atherosclerotic lesions by Masson trichrome staining. n = 9 mice per group. Mann-Whitney test was conducted to determine statistical significance between the two groups. ns, no significant difference.

To study the role of Axl in bone marrow-derived cells in advanced atherosclerosis, we generated chimeric mice by transplanting *Axl*^*−/−*^ bone marrow cells into lethally irradiated *Ldlr*^*−/−*^ mice. All mice were on the C57BL/6 J background, and control mice received bone marrow from wild-type littermates. Six weeks after transplantation, the mice were fed the Western-type diet for an additional 17 weeks. The two groups of mice gained weight equally and had similar metabolic parameters including plasma cholesterol, fasting blood glucose, and insulin (Supplementary Figure IA–D) and similar immune cell subset distribution in the peripheral blood (Supplementary Figure IE). We confirmed the successful repopulation of donor cells in the recipient mice by observing the loss of Axl expression in splenic dendritic cells of mice receiving bone marrows from *Axl*^*−/−*^ donors (Supplementary Figure IF). We first asked whether Axl was expressed in the aortic root lesional cells of the control group and, if so, whether this expression was successfully suppressed in the lesions of the chimeric mice. Indeed, Axl immunostaining was clearly evident in the control lesions but not in the *Axl*^*−/−*^ → *Ldlr*^*−/−*^ lesions, and the pattern of expression was similar to that of F4/80^+^ macrophages (Supplementary Figure IG). These data are consistent with the recent finding that Axl expression is induced in cultured macrophages under inflammatory conditions^8^. We next quantified the overall lesion area and necrotic area of the aortic root plaques of WT → *Ldlr*^*−/−*^ and *Axl*^*−/−*^ → *Ldlr*^*−/−*^ mice and found no statistical difference between the two groups (Fig. 1A–C). Moreover, the percent distribution of lesional macrophages, dendritic cells, and smooth muscle cells was similar between the two groups of mice (Fig. 1D). A previous study demonstrated that deficiency of Gas6, which is a ligand that signals via the TAM family of receptors, was associated with increased collagen deposition and a more stable plaque^9^. However, hematopoietic cell-Axl deficiency did not result in significant differences in intimal collagen content or fibrous cap thickness (Fig. 1E).

**Figure 2:**
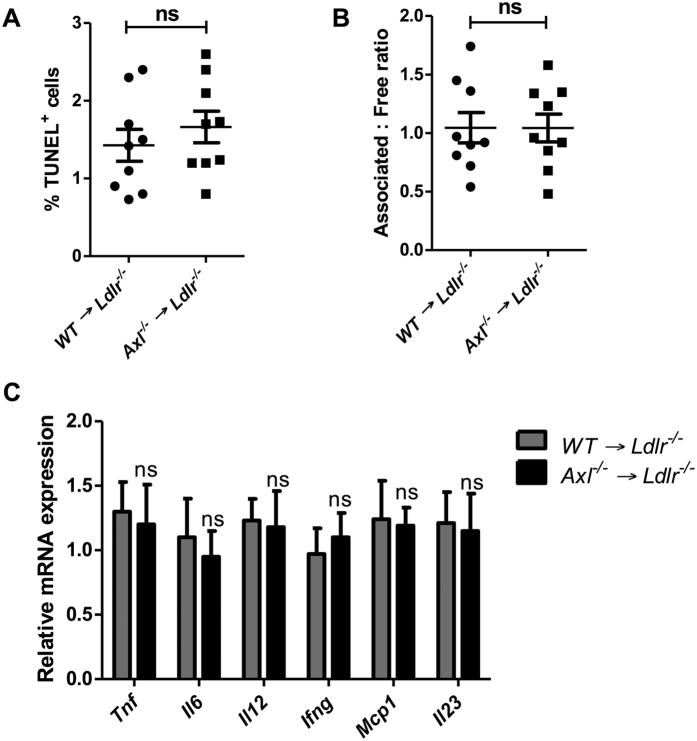
Hematopoietic cell-Axl deficiency does not affect advanced atherosclerosis apoptosis, efferocytosis, or inflammatory gene expression. (**A**) Fluorescence microscopy-based quantification of TUNEL^+^ cells as a percentage of total lesional cells in WT → *Ldlr*^*−/−*^ or *Axl*^*−/−*^ → *Ldlr*^*−/−*^ mice fed the WD for 17 weeks. (**B**) *In-situ* efferocytosis analysis of aortic root sections; the ratio of macrophage-associated TUNEL^+^ cells to free TUNEL^+^ cells is a measure of lesional efferocytosis. (**C**) Quantitative RT-PCR for the indicated lesional mRNAs. n = 9 mice per group. Mann-Whitney test was conducted to determine statistical significance between the groups. ns, no significant difference.

In view of the pro-survival role of Axl signaling in several cell types, we analyzed whether deficiency of Axl increases lesional cell apoptosis. As demonstrated in Fig. 2A, the extent of TUNEL staining, a reliable measure of apoptosis in atherosclerotic lesions, was similar between control and hematopoietic cell -Axl deficient mice. Similar data was obtained with analysis of expression of cleaved caspase-3, another marker for apoptotic cells (Supplementary Figure II). Furthermore, Axl is a known efferocytosis receptor in macrophages and dendritic cells^8^,^10^, but there was no significant difference in the *in-situ* efferocytic index of WT → *Ldlr*^*−/−*^ versus *Axl*^*−/−*^ → *Ldlr*^*−/−*^ lesions (Fig. 2B and Supplementary Figure III). Because lesional cell apoptosis coupled to defective efferocytosis contributes to necrotic core formation^3^, these data are consistent with the similar lesional necrotic area between the two groups of mice.

Axl-Gas6 signaling is known to elicit an anti-inflammatory response in innate immune cells via activation of SOCS family of proteins^11^. Thus, we tested whether expression of pro-inflammatory markers was increased in the atherosclerotic lesions of hematopoietic cell -Axl deficient mice. Consistent with all of the lesional data thus far, the expression levels of *Tnf, Il6, Il12, Ifng, Mcp1*, and *Il23* was similar between the two groups of mice (Fig. 2C). We then turned our attention to T cells, because activated effector T cells promote atherosclerosis progression, and T cell-derived Protein S acts on TAM receptors on dendritic cells to dampen the magnitude of the T cell response^12^. However, we did not observe changes in the levels of effector T cells in the spleens of *Axl*^*−/−*^ → *Ldlr*^*−/−*^ mice (Supplementary Figure IV).

**Figure 3:**
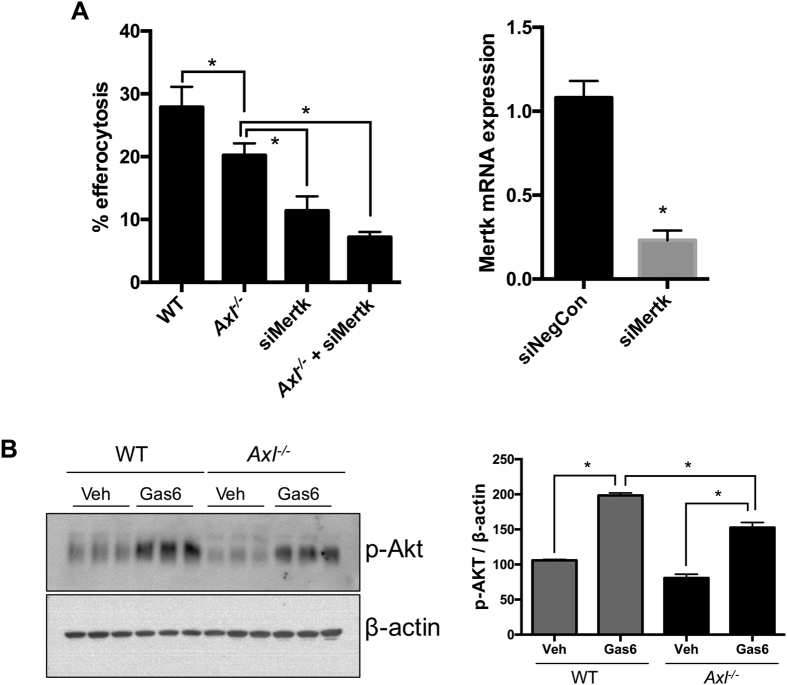
Axl deficiency is not compensated functionally by Mertk expression. (**A**) WT and *Axl*^*−/−*^ BMDMs were transfected with either negative control siRNA or Mertk siRNA as indicated. 48 h post-transfection, these macrophages were incubated with fluorescently-labeled apoptotic jurkat cells at a ratio of 1:10 for 60 min. Percent efferocytosis was analyzed by fluorescence microscopy. *p < 0.05 by ANOVA and Turkey’s multiple comparison test. The bar graph on the right demonstrates the efficiency of siRNA mediated knockdown of Mertk in BMDMs as determined by real-time PCR. *p < 0.05 by Student’s t-test. (**B**) WT and *Axl*^*−/−*^ BMDM were treated with cell culture supernatants of γ-carboxyated human Gas-6-expressing HEK293-6E cells (Gas6) or supernatants from control HEK293-6E cells (Veh) for 30 min as indicated. Whole cell lysates were immunblotted for phospho-AKT. The bar graph represents densitometric quantification of the immunoblot normalized to the expression of β-actin. The data were derived from triplicates of the experiment. *p < 0.05 by ANOVA and multiple comparison test.

Finally, we tested whether the lack of effect on inflammation, apoptosis, and efferocytosis seen in the hematopoietic cell -Axl deficient mice was due to a compensatory effect of increased expression of other TAM family members, namely, MerTK and Tyro3 in macrophages. However, immunostaining demonstrated that the expression levels of MerTK in the *Axl*^*−/−*^ lesional macrophages was similar to WT (Supplementary Figure VA). The expression levels of Tyro3 on lesional macrophages was very low but similar between WT and BM-*Axl*^*−/−*^ mice (Supplementary Figure VB). Also, the loss of Axl did not result in a compensatory increase in efferocytosis (Fig. 3A) or Gas6-mediated pAKT signaling (Fig. 3B) through Mertk and Tyro3 in bone-marrow derived macrophages. These combined data show that Axl expression in bone marrow-derived cells does not play a major role in affecting the progression of advanced atherosclerotic lesions in WD-fed *Ldlr*^*−/−*^ mice.

## Discussion

Identifying the molecular-cellular processes involved in advanced atherosclerotic lesion progression is critical for understanding how clinically important plaques form and how their development may be therapeutically suppressed. In this context, TAM receptors, particularly MerTK and Axl, are of interest because of their expression in lesional cells and their involvement in both efferocytosis and inflammation control^4^,^5^,^6^,^7^,^11^, both of which go awry in advanced atherosclerosis. While previous studies have clearly demonstrated a protective role for MerTK in advanced atherosclerosis^6^,^7^, including in a bone-marrow chimera model^7^ similar to that used in this study, the role of Axl was not known. Our data showing that Axl deficiency in bone marrow-derived cells does not affect the most important parameters of advanced atherosclerosis is quite surprising and cannot be explained by compensatory up-regulation of MerTK or Tyro3. These findings are in contrast to a protective effect of holo-deficiency of Axl in intima media thickening (IMT) induced by partial ligation of carotid artery in mice^13^. The differences could be due to the dominant role of endothelial and smooth muscle cells in the pathogenesis of injury-induced IMT in contrast to the major role of myeloid cells in the pathogenesis of classical atherosclerosis. Whether Axl signaling in endothelial and smooth muscle cells play a role in atherosclerosis progression will require a future study. However, it is important to note that human genetic studies have revealed that SNPs in Gas6 and Axl are not associated with carotid atherosclerosis^14^.

In the present study, our focus was predominantly on understanding the role of Axl expressed on hematopoietic-derived cells in advanced atherosclerosis and thus we chose to use the *Axl*^*−/−*^ bone marrow chimera model. *Ldlr*^*−/−*^ mice are used for bone marrow transplant studies, because reconstitution of *Apoe*^*+/+*^ donors into *Apoe*^*−/−*^ recipients results in lowering of cholesterol levels and suppression of atherosclerosis^15^. In addition, atherosclerosis in Western diet-fed *Ldlr*^*−/−*^ mice is driven by LDL, as in humans, and this model also avoids the confounding effect of basal apolipoprotein E in various immune cells, which could impact atherosclerosis progression^16^,^17^,^18^.

Consistent with a previous report showing that Axl is induced by treatment of cultured macrophages with inflammatory stimuli^8^, we found that macrophages in inflammatory advanced atherosclerotic lesions express Axl. That report also showed that efferocytosis is partially defective in bone marrow-derived macrophages isolated from *Axl*^*−/−*^ mice and that mice treated with an Axl-activating antibody have a suppressed response to LPS *in vivo*^8^. In contrast, efferocytosis and inflammation were similar in the advanced atherosclerotic lesions of *Axl*^*−/−*^ → *Ldlr*^*−/−*^ and WT → *Ldlr*^*−/−*^ mice. This finding, combined with previous reports showing that MerTK deficiency affects both efferocytosis and inflammation in lesions^6^,^7^ suggests that MerTK is the dominant TAM receptor in these processes in the setting of atherosclerosis. The mechanism for MerTK dominance remains to be elucidated and could in theory be related to differences in the expression levels of the two receptors and/or in downstream signaling mechanisms. In addition, if the TAM receptor ligand Protein S were particularly important in advanced atherosclerosis, the finding that Protein S has a greater affinity for MerTK versus Axl may help explain the dominance of MerTK in this setting^19^. In the context of these possibilities, a study showed that advanced human carotid arteries were enriched in MerTK when compared with normal carotid arteries and that Protein S levels increased with advanced plaque grade^20^. While both Axl and Mertk are considered to be involved in efferocytosis and suppression of inflammation, a recent report raises the possibility that they may play opposing roles in certain inflammatory settings, such as that seen in a mouse model of nephrotoxic serum-induced glomerulonephritis^21^. Because that study was conducted using holo-Axl KO and holo-Mertk KO, the specific cell types that are responsible for these opposing effects remain to be elucidated.

In summary, in stark contrast to the findings with MerTK deficiency^6^,^7^, hematopoietic cell-Axl deficiency in WD-fed *Ldlr*^*−/−*^ mice does not affect the progression of advanced atherosclerosis or lesional processes associated with TAM receptor signaling. These findings suggest a heretofore unappreciated TAM receptor functional hierarchy in advanced atherosclerosis. Although the mechanistic basis for this hierarchy and its implications for atherosclerotic heart disease remain to be explored, the data highlight new issues related to the biology of these receptors in an actual disease setting driven by chronic, maladaptive inflammation.

## Materials and Methods

### Animals, bone marrow transplantation, and diet

*Axl*^*−/−*^ mice on a C57BL6 background (11 backcrosses) were bred and maintained in the animal facility at Columbia University. 8 week old female *Ldlr*^*−/−*^ mice (Stock # 002207) were purchased from Jackson laboratories. *Ldlr*^*−/−*^ mice were lethally irradiated (10 Gy) and transplanted with bone marrow cells isolated from either *Axl*^*−/−*^ or WT littermate control mice. 6 weeks post-transplantation, the mice were fed the Western type diet ab libitum (TD88137, Teklad Diets, Envigo) for 17 weeks. All animal experiments were conducted following relevant guidelines and regulations and approved by the Institutional Animal Care and Use Committee of Columbia University.

### Metabolic parameter measurements

Animals were weighed weekly and the weights recorded. For measurements of plasma glucose and insulin, the mice were fasted for 5 hours before blood draw. Plasma glucose was measured using glucose test strips (Accu-Chek) and standard glucometer. Plasma insulin was measured using ELISA (Crystal Chem). Plasma cholesterol was measured using Cholesterol E kit (Wako) following manufacturer’s instructions.

### Atherosclerotic lesion measurements

Aortic root atherosclerotic sections were stained with hematoxylin and eosin and total lesion area and necrotic area were measured by a person blinded to the groups as described previously^6^. Briefly, using ImageProPlus, the total intimal area from 6 sections per mouse was measured to arrive at the total lesion area. For necrotic area measurements, boundary lines were drawn around areas lacking hematoxylin-stained nuclei (acellular), which were then quantified using a 3,000-μm^2^ threshold to avoid including regions that did not represent substantial areas of necrosis. To measure collagen content of the lesions, 3 sections per mouse were stained with Masson’s trichrome stain following manufacturer’s instructions. An electronic color threshold was applied on the blue stained regions of the plaque (collagen) using ImageJ, and the percent collagen positive area was then determined.

### TUNEL, immunostaining, and *in-situ* efferocytosis assay

Lesional cell apoptosis was measured by terminal deoxynucleotidyl transferase dUTP nick end labeling (TUNEL) assay using TMR red *in-situ* cell death detection kit (Roche) following manufacturer’s instructions. The TUNEL stained aortic root sections were imaged under a fluorescence microscope and quantification was conducted using ImageJ image analysis software. *In-situ* efferocytosis assay was conducted as described previously^6^. Briefly, aortic root sections were stained with TUNEL followed by immunostaining for macrophages using anti-F4/80 antibody (AbD Serotech). Lesional efferocytosis was evaluated by counting the number of free versus macrophage-associated TUNEL^+^ cells in atherosclerotic lesional sections. Apoptotic cells were considered “free” when they were not surrounded by or in contact with macrophages.

### mRNA isolation and real-time PCR

Aortic root sections were lysed in RLT buffer containing β-mercaptoethanol following which RNA was isolated using RNeasy Micro kit (Qiagen). cDNA was generated from the isolated mRNA using Superscript Vilo cDNA synthesis kit. Gene expression analysis was conducted by quantitative real time PCR (Applied Biosystems 7500 RTPCR machine) using SYBR green probe. The following primers were used in this study: TNF-α (5′-CATCTTCTCAAAATTCGAGTGACAA-3′/5′-TGGGAGTAGA CAAGGTACAACCC-3′); IL-12 p40 (5′-CCTGCATCTAGAGGCTGTCC-3′/5′-GGCAAACCAGGAGAT GGTTA-3′); MCP-1 (5′-CCCCACTCACCTGCTGCTACT-3′/5′-TTTACGGGTCAACTTGACATTC-3′); Mertk (5′-GTGGCAGTGAAGACCATGAAGTTG-3′/5′-GAACTCCGGGATAGGGAGTCAT-3′). The primers for IL-6, IFN-γ, and IL-23 were obtained from Qiagen.

### Statistics

The data presented in this manuscript are represented as means ± SEM. The n numbers for each group are indicated in the figure legends. The statistical test conducted to determine significance for each experiment is indicated in the figure legends. A p-value of <0.05 was considered as statistically significant.

## Additional Information

**How to cite this article**: Subramanian, M. *et al*. Deficiency of AXL in Bone Marrow-Derived Cells Does Not Affect Advanced Atherosclerotic Lesion Progression. *Sci. Rep.* **6**, 39111; doi: 10.1038/srep39111 (2016).

**Publisher's note:** Springer Nature remains neutral with regard to jurisdictional claims in published maps and institutional affiliations.

## Supplementary Material

Supplementary Figures
