# Supplementary material for: Deficiency of AXL in Bone Marrow-Derived Cells Does Not Affect Advanced Atherosclerotic Lesion Progression
Source: Sci Rep. 2016 Dec 13;6:39111. doi: 10.1038/srep39111 (PMC5153620; doi:10.1038/srep39111)
Supplement: Supplementary Figures [file srep39111-s1.pdf]

Supplementary Information

Deficiency of AXL in Bone Marrow-Derived Cells Does Not Affect Advanced Atherosclerotic Lesion Progression

Manikandan Subramanian, Jonathan D. Proto, Glenn K. Matsushima, Ira Tabas

Figure I

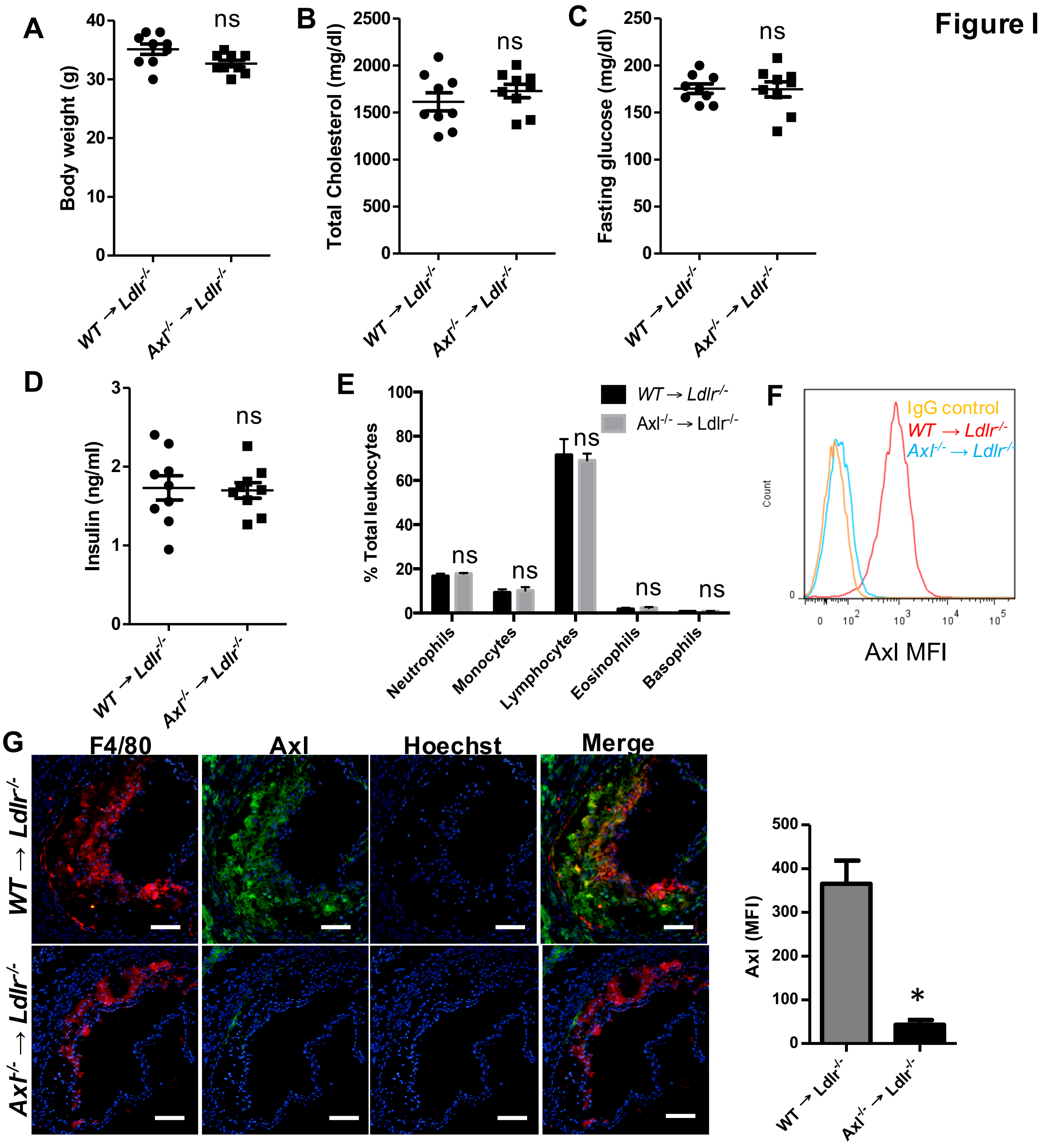

Supplementary Figure I.
Measurement of body weight (**A**), plasma cholesterol (**B**), 5-h fasting blood glucose (**C**), and plasma insulin (**D**) in WT → *Ldlr*$^{-/-}$ or *Axl*$^{-/-}$ → *Ldlr*$^{-/-}$ mice fed the WD for 17 weeks. n = 9 mice per group. Mann-Whitney test was conducted to determine statistical significance. ns, no significant difference. (**E**) Differential WBC count of WT → *Ldlr*$^{-/-}$ or *Axl*$^{-/-}$ → *Ldlr*$^{-/-}$ mice fed the WD for 17 weeks. (**F**) Representative flow-cytometry histogram of cell surface Axl expression in splenic dendritic cells (CD11c+) of WT → *Ldlr*$^{-/-}$ or *Axl*$^{-/-}$ → *Ldlr*$^{-/-}$ mice fed the WD for 17 weeks. (**G**) Representative fluorescence microscopic images of aortic root atherosclerotic lesional sections immunostained for F4/80 and Axl and counterstained with Hoechst nuclear dye, with quantification based on mean fluorescence intensity (MFI) of Axl in F4/80^{+} regions of the lesions. n = 5 mice per group. *, $p < 0.05$ as determined by Student's t-test. Bar, 100 µm.

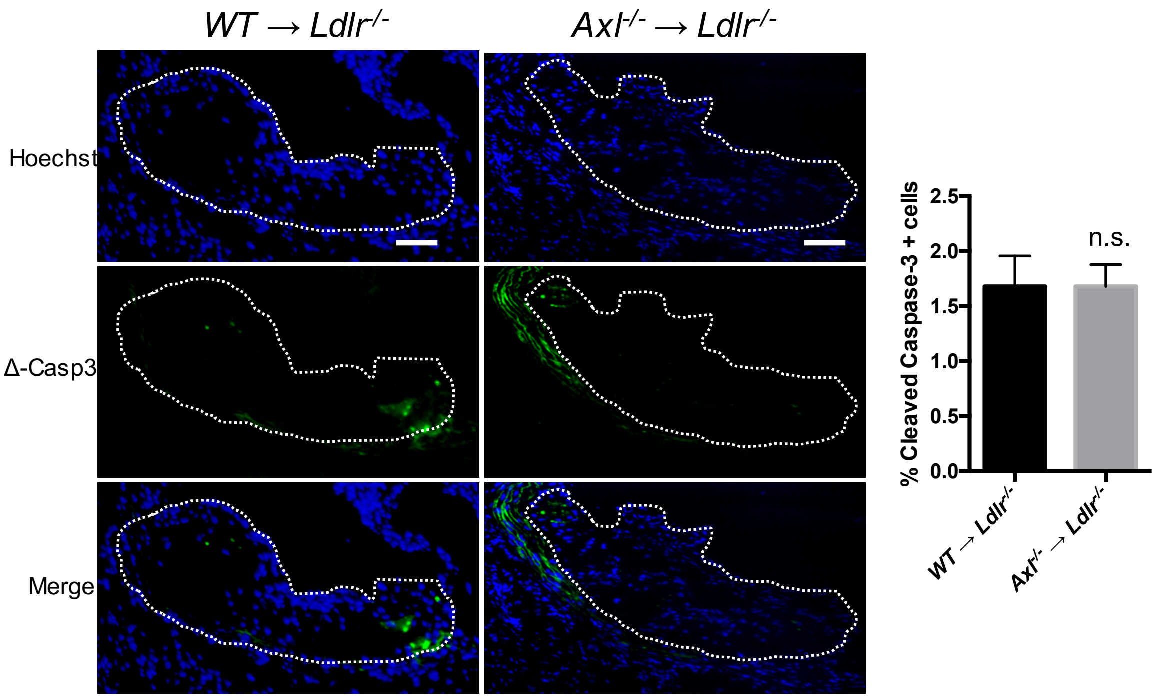

Supplementary Figure II.
Aortic root sections of 17 week WD fed WT → *Ldlr*$^{-/-}$ and *Axl*$^{-/-}$ → *Ldlr*$^{-/-}$ mice were immunostained with antibody that specifically recognizes the cleaved form of caspase-3 (green). Nucleus was counterstained with Hoechst 33342 (blue). The white dotted lines demarcate intimal lesion. Bar, 100 μm. n = 9 mice per group. ns, no significant difference as determined by Mann-Whitney test.

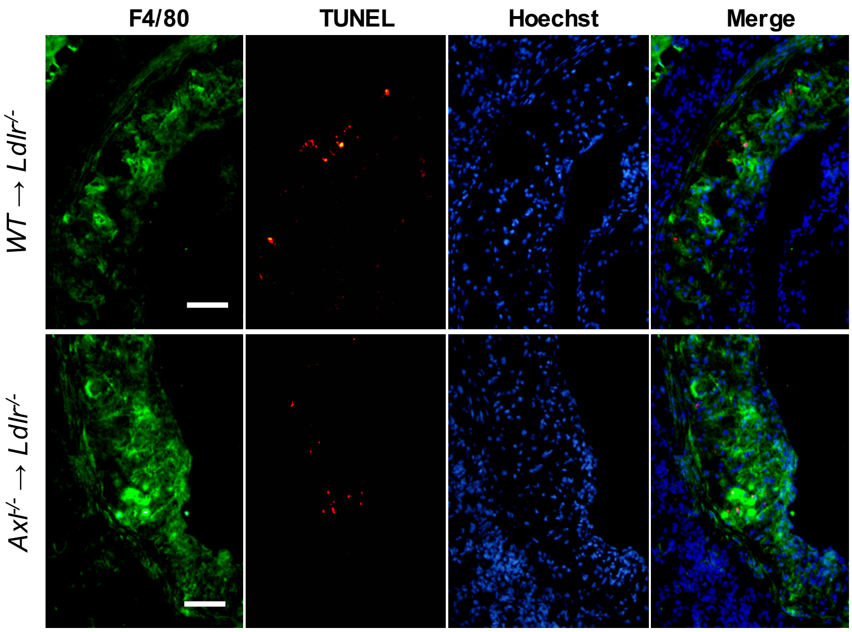

Supplementary Figure III.
In-situ efferocytosis assay was conducted by TUNEL staining (red) of aortic root sections of 17 week WD fed WT → *Ldlr*$^{-/-}$ and *Axl*$^{-/-}$ → *Ldlr*$^{-/-}$ mice followed by immunostaining with antibody that specifically recognizes F4/80 (green). Nucleus was counterstained with Hoechst 33342 (blue). Bar, 100 μm.

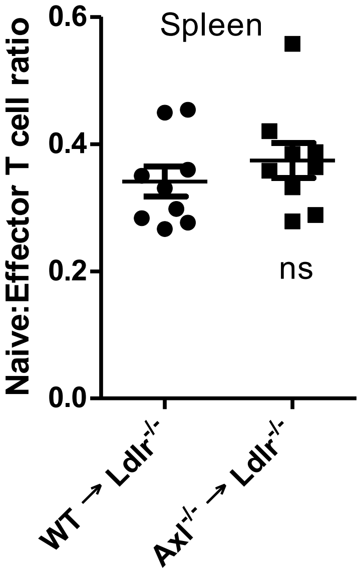

Supplementary Figure IV.
Flow cytometry-based analysis of the ratio of naïve:effector T cells in the spleens of WT → *Ldlr*$^{-/-}$ or *Axl*$^{-/-}$ → *Ldlr*$^{-/-}$ mice fed the WD for 17 weeks. CD3^{+} cells that were CD44^{lo}CD62L^{hi} were classified as naïve, whereas CD44^{hi}CD62L^{lo} were classified as effector T cells. The were no significant difference between the two cohorts.

Supplementary Figure V

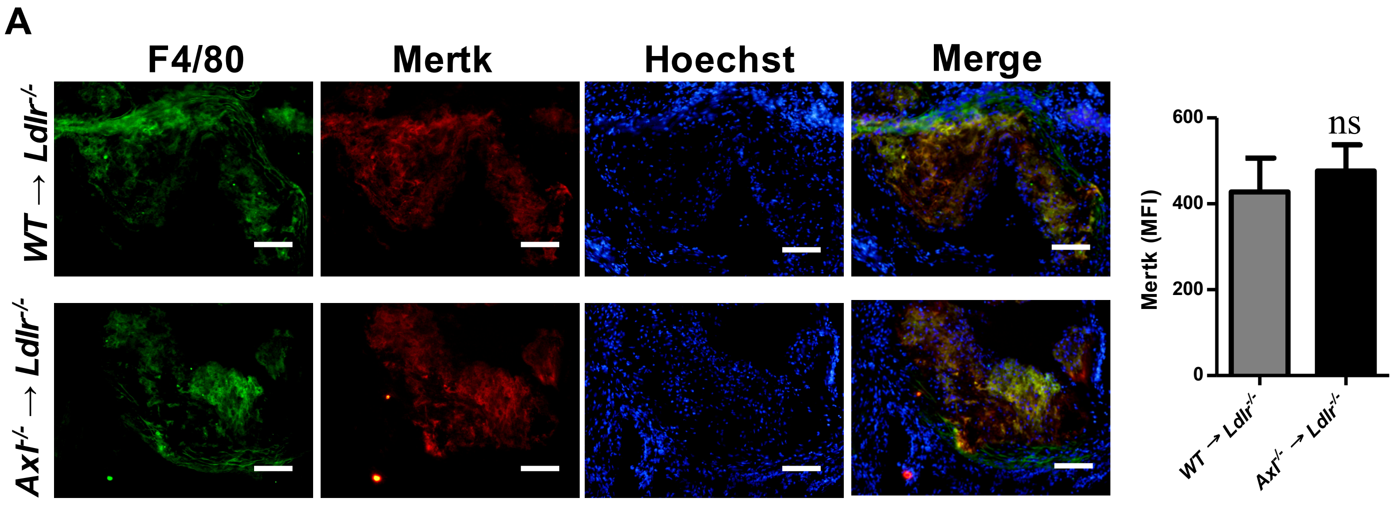

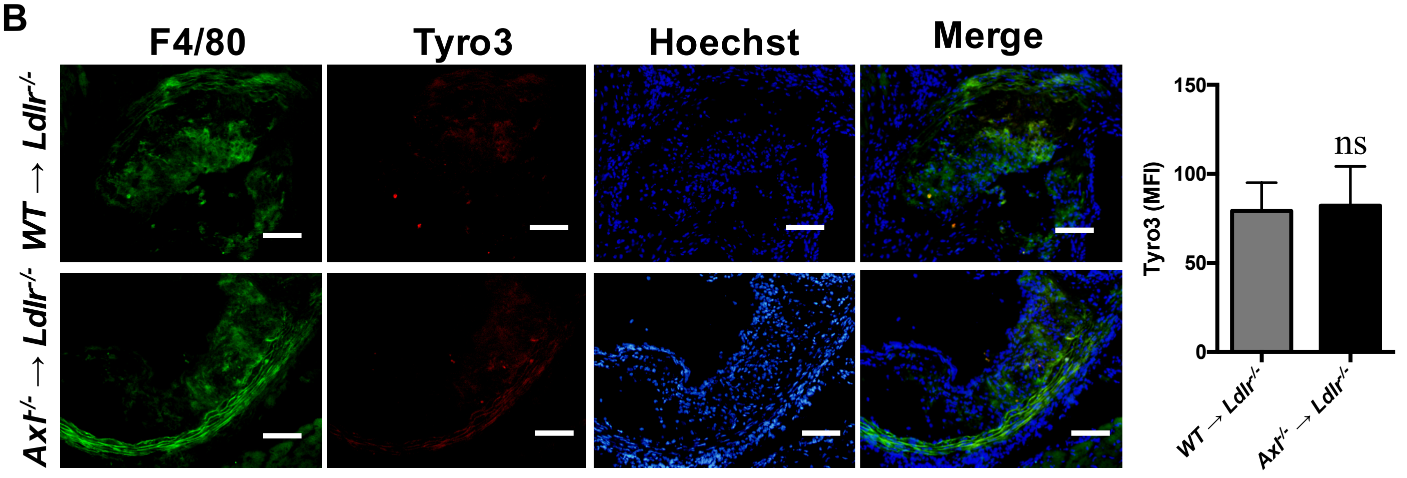

Supplementary Figure V.
Representative fluorescence microscopic images of aortic root atherosclerotic lesional sections immunostained for F4/80 and MerTK (top panel) or F4/80 and Tyro3 (bottom panel). The expression levels of Mertk and Tyro3 was quantified based on mean fluorescence intensity (MFI) of MerTK and Tyro3 respectively in F4/80$^+$ regions of the intimal lesion. n = 5 mice per group. ns, no significant difference as determined by Student's t-test. Bar, 100 µm.
